# High prevalence of oxacillinases in clinical multidrug-resistant *Acinetobacter baumannii* isolates from the Tshwane region, South Africa – an update

**DOI:** 10.1186/s12879-015-1246-8

**Published:** 2015-11-14

**Authors:** Michelle Lowings, Marthie Magdaleen Ehlers, Andries William Dreyer, Marleen Magdalena Kock

**Affiliations:** Department of Medical Microbiology, University of Pretoria, Pretoria, South Africa; National Health Laboratory Service, Tshwane Academic Division, Pretoria, South Africa; Centre for Tuberculosis, National Institute for Communicable Diseases, Johannesburg, South Africa

**Keywords:** *Acinetobacter baumannii*, Beta-lactamases, M-PCR, PFGE, MLST

## Abstract

**Background:**

*Acinetobacter baumannii* is an important hospital-acquired pathogen in healthcare facilities that frequently causes bacteraemia and ventilator-associated pneumonia in intensive care units. *Acinetobacter baumannii* can be isolated from various sites in the hospital environment like medical equipment, bed linen, medical personnel and indwelling catheters. It is difficult to treat *A. baumannii* infections because of their highly resistant antimicrobial profiles. The purpose of this study was to determine the prevalence of β-lactamase genes in multidrug-resistant (MDR) clinical *A. baumannii* isolates using Multiplex-PCR (M-PCR) assays.

**Methods:**

One hundred MDR *A. baumannii* isolates were collected from the diagnostic division of the Department of Medical Microbiology after routine analysis of the submitted specimens. All collected isolates were identified and tested for susceptibility using the VITEK 2® system (bioMérieux, France). Six isolates were excluded from this study because the isolates were incorrectly identified as *A. baumannii* with the VITEK 2® system (bioMérieux, France). Molecular tests, namely M-PCR assays, pulsed-field gel electrophoresis (PFGE) and multilocus sequence typing (MLST) were performed. MLST analyses were performed on representative isolates from the four major pulsotypes (≥5 isolates with 80 % similarity) and selective isolates from each minor pulsotype.

**Results:**

All the *A. baumannii* isolates showed 100 % resistance to ampicillin, amoxicillin, cefuroxime, cefuroximine axetil, cefoxitin, cefotaxime and nitrofurantoin. Seven percent of the isolates were resistant to amikacin. Two percent of the isolates were classified as having intermediate susceptibility to tigecycline. *A. baumannii* isolates showed an antibiotic resistance profile of 67 % and higher to antibiotics, such as ceftazidime, cefepime, imipenem, meropenem, gentamicin, ciprofloxacin and trimethoprim/sulfamethoxazole. None of the isolates were resistant to colistin. The M-PCR assays showed that 99 % of the isolates contained the OXA-51 gene and 77 % contained the OXA-23 gene. None of the isolates contained the GES, GIM, IMP, KPC, NDM, OXA-24, OXA-58, PER, SIM, SPM, VEB and VIM genes. Representative *A. baumannii* isolates were grouped into five existing sequence types (ST): ST106, ST258, ST339, ST502, ST758 and ST848. Isolates belonging to the pan-European clonal lineages I and II (EUI and EUII) were identified.

**Conclusion:**

The high prevalence of MDR *A. baumannii* isolates has a severe impact on available treatment choices and this in return impacts on treatment outcomes in the studied healthcare facilities. The most dominant ST among the collected isolates was ST758, member of the EUI group. The presence of the OXA-23 gene was not restricted to a specific ST. Continuous research and surveillance is necessary to monitor the circulating β-lactamase genes in clinical settings to guide infection control policies in order to try and curb the spread of this bacterium.

## Background

*Acinetobacter baumannii* is an emerging opportunistic pathogen that can become resistant to multiple antimicrobial agents [1–3]. Over the past 20 years *A. baumannii*’s clinical importance has been driven by its ability to obtain antimicrobial resistance factors [4, 5]. Resistance is acquired through the transfer of integrons, plasmids or transposons that carry groups of genes encoding resistance to several antibiotic families [6]. This bacterium can survive under a wide range of environmental conditions and on surfaces for extended periods of time and this can lead to both endemicity and outbreaks in healthcare facilities [4, 7].

Major problems are caused by this pathogen, especially among critically ill hospitalised patients in intensive care units (ICU’s) [5, 6, 8]. Clinical manifestations that can be associated with *A. baumannii* include: bacteraemia, meningitis, pneumonia, urinary tract infection, ventilator-associated pneumonia (VAP) and wound infections [8–10]. Patients with underlying chronic disease, decreased immunity, indwelling catheters and prolonged hospitalisation are more at risk to become colonised and develop subsequent infection with *A. baumannii* [7–9, 11].

This pathogen possesses different antimicrobial resistance mechanisms, which include the enzymatic inactivation of β-lactam antibiotics (cephalosporins, carbapenems and monobactams) through the production of extended spectrum β-lactamases, carbapenemases and AmpC-type (ampicillin class C) enzymes [12–15]. The β-lactamases of Gram-negative bacteria belong to Ambler classes A to D [12, 13]. The inherent class D oxacillinases (OXA) of *A. baumannii* belong to the chromosomally encoded OXA-51-like enzyme group [16–18]. However, the OXA-51 enzyme has been under considerable pressure due to antibiotic use and has evolved and now plays an important role in the carbapenem resistance of *A. baumannii* isolates [19]. If IS*Aba*I is located upstream of the *bla*_OXA_ genes the *A. baumannii* isolate will have an increased OXA-51 expression which leads to carbapenem resistance (imipenem and meropenem) [19–21].

Infections caused by *A. baumannii* are difficult to treat because of the resistance to antimicrobial agents, especially β-lactams [3, 22]. There have been reports of MDR *A. baumannii* from hospitals all around the world (Argentina, Brazil, China, Europe and the United Kingdom) [4, 23, 24]. Only a few antimicrobial agents namely colistin (in combination with carbapenems) and tigecycline are available for the treatment of MDR *A. baumannii* [1, 2, 15]. However, there are now reports of colistin resistance in *A. baumannii* isolates, which is of great concern as colistin plays a pivotal role in MDR *A. baumannii* treatment [25–28]. Fortunately colistin resistance in *A. baumannii* is still rare [21, 25].

The aim of this study was to detect the prevalence of β-lactamase genes and to determine the genetic relationship among the *A. baumannii* isolates circulating in our clinical setting. The prevalence of β-lactamase genes is important for the surveillance of circulating *A. baumannii* strains as it provides more information for the infection control policies of the hospitals.

## Methods

### Study design and setting

The quantitative study was aimed to determine the prevalence of β-lactamase genes in consecutively collected *A. baumannii* isolates obtained from clinical specimens and to determine the genetic relatedness of circulating strains. The study was conducted at the Department of Medical Microbiology, Prinshof Campus, University of Pretoria/National Health Laboratory Service (NHLS) and received ethical approval from the Research Ethics Committee, Faculty of Health Sciences, University of Pretoria (121/2013); a waiver for informed consent was provided.

### Collection, culturing and storage of *A. baumannii* clinical isolates

One hundred consecutive clinical *A. baumannii* isolates were collected without prior knowledge of patient information, hospital ward, specimen type and antibiotic susceptibility status. All 100 *A. baumannii* isolates were collected from the diagnostic laboratory during May to July 2013. The laboratory processes specimens from a tertiary academic hospital as well as district hospitals and various clinics as part of standard care. Routine analysis was performed as requested on the form and according to sample type. The isolates were identified and tested for susceptibility using the automated VITEK® 2 system (BioMérieux, France) with the VITEK® 2 GN card and the VITEK® 2 AST-N255 card. Gender, age, type of specimen, hospital, ward and the antibiotic susceptibility profiles were collected.

One colony of an isolate (obtained from a blood agar plate) was inoculated into 5 ml tryptone soya broth (TSB) (Oxoid, UK) and incubated on a shaking incubator (Vacutec, South Africa) at 35°C for a period of 18 to 24 h. Growth was indicated by turbidity in the broth. Isolates were stored by adding 900 μl of 50% glycerol (Merck, Germany) to 900 μl of the overnight TSB culture (Oxoid, UK) in 2 ml cryotubes (Greiner Bio-One, Germany) in a one to one ratio. This was done in triplicate and the isolates were stored in a -80°C freezer (New Brunswick, USA). The quality control strains used were the multidrug resistant *A. baumannii* ATCC® BAA-1605 strain; *Klebsiella pneumoniae* ATCC 8303 and *K. pneumoniae* ATCC BAA-2146.

### DNA extraction of *A. baumannii* isolates

The genomic DNA of each of the 100 *A. baumannii* isolates was extracted from a single colony grown in TSB (Oxoid, UK) culture media by using the ZR Fungal/Bacterial kit [29]. The manufacturer’s instructions were followed with some modifications; instead of 50 to 100 mg of cells, 1 000 μl of overnight broth was used, centrifuged (Eppendorf 5417C, Germany) and the pellet was resuspended in phosphate buffered saline (PBS) solution (pH 7.2, Gibco® PBS, Life Technologies Corporation, USA) and 600 μl of the lysis solution were used instead of the prescribed 750 μl. The extracted genomic DNA was stored at -20 °C (Defy Ltd, Multimode, SA) until further analysis.

### The detection of β-lactamase genes in *A. baumannii* by using M-PCR assays

Multiplex PCR assays were performed by using the QIAGEN® M-PCR kit (QIAGEN®, Germany) for the detection of several antibiotic resistance genes in *A. baumannii* (Table 1). An initial denaturation step of 95 °C for 15 min, followed by 30 cycles of denaturation at 94 °C for 30 s, an annealing temperature dependent on the melting temperature of the primer pair (multiplex I: 52 °C; multiplex II and III: 57 °C and multiplex IV: 60 °C) and extension at 72 °C for 90 s, followed by the final extension step at 70 °C for 10 min. A negative control (sterile ultrapure water) was included for all M-PCR assays. The following positive controls were used for multiplex I to IV: *A. baumannii* ATCC® BAA-1605™ strain; *K. pneumoniae* ATCC 8303 [*Klebsiella pneumoniae* carbapenemase (KPC) positive control] and *K. pneumoniae* ATCC BAA-2146 [New Delhi metallo-β-lactamase (NDM) positive control]. All isolates testing negative for the presence of the inherent OXA-51 gene were sent for identification using matrix assisted laser desorption time of flight mass spectrometry (MALDI-TOF).

**Table 1:** Oligonucleotide primer sequences used for the detection of β-lactamases in four Multiplex PCR assays of *A. baumannii* clinical isolates

Target	Primer name	Primer sequence (5’ to 3’)^a^	Amplicon size (bp)	Reference
Multiplex I:				
OXA-23	*bla*_OXA-23_ (F)	GATCGGATTGGAGAACCAGA	501	[51]
	*bla*_OXA-23_ (R)	ATTTCTGACCGCATTTCCAT		
OXA-24	*bla*_OXA-24_ (F)	GGTTAGTTGGCCCCCTTAAA	246	
	*bla*_OXA-24_ (R)	AGTTGAGCGAAAAGGGGATT		
OXA-51	*bla*_OXA-51_ (F)	TAATGCTTTGATCGGCCTTG	353	
	*bla*_OXA-51_ (R)	TGGATTGCACTTCATCTTGG		
OXA-58	*bla*_OXA-58_ (F)	AAGTATTGGGGCTTGTGCTG	599	
	*bla*_OXA-58_ (R)	CCCCTCTGCGCTCTACATAC		
Multiplex II:				
VIM	*bla*_VIM_ (F)	GATGGTGTTTGGTCGCATA	390	[52]
	*bla*_VIM_ (R)	CGAATGCGCAGCACCAG		
KPC	*bla*_KPC_ (F)	CATTCAAGGGCTTTCTTGCTGC	538	
	*bla*_KPC_ (R)	ACGACGGCATAGTCATTTGC		
IMP	*bla*_IMP_ (F)	TTGACACTCCATTTACDG^b^	139	
	*bla*_IMP_ (R)	GATYGAGAATTAAGCCACYCT		
Multiplex III:				
GES	*bla*_GES_ (F)	AGTCGGCTAGACCGGAAAG	399	[52]
	*bla*_GES_ (R)	TTTGTCCGTGCTCAGGAT		
PER	*bla*_PER_ (F)	GCTCCGATAATGAAAGCGT	520	
	*bla*_PER_ (R)	TTCGGCTTGACTCGGCTGA		
VEB	*bla*_VEB_ (F)	CATTTCCCGATGCAAAGCGT	648	
	*bla*_VEB_ (R)	CGAAGTTTCTTTGGACTCTG		
Multiplex IV:				
GIM	*bla*_GIM_ (F)	CGTTGCCAGCTTTAGCTCAGG	279	[53]
	*bla*_GIM_ (R)	GCAACTTGATACCAGCAGTGCG		
SPM	*bla*_SPM_ (F)	GGGTGGCTAAGACTATGAAGCC	447	
	*bla*_SPM_ (R)	GCCGCCGAGCTGAATCGG		
SIM-1	*bla*_SIM_ (F)	TTGCGGAAGAAGCCCAGCCAG	613	
	*bla*_SIM_ (R)	GCGTCTCCGATTTCACTGTGGC		
NDM – 1	*bla*_NDM-1_(F)	CCCGGCCACACCAGTGACA	129	
	*bla*_NDM-1_(R)	GTAGTGCTCAGTGTCGGCAT		

^a^All oligonucleotides were synthesised and purified by Inqaba Biotechnical Industries, South Africa

^b^Y = T or C; D = A or G or T

### Analysis of the Multiplex PCR products

A 1.8 % (m/v) MetaPhor agarose gel (Lonza, USA) with 5 μl of ethidium bromide (EtBr) (10 μg.ml^−1^) (Promega, USA) was used to visualise the PCR products after gel electrophoresis (Bio-Rad, USA). The 1.8 % (m/v) MetaPhor® agarose gel was prepared according to the manufacturer’s instructions (Lonza, USA).

A ready-to-use 100 bp DNA ladder (Fermentas, ThermoScientific, USA) was used to determine the approximate sizes of the amplicons. Gel electrophoresis (Bio-Rad, Germany) was performed at 90 V for 100 min with 1x Tris-Borate-EDTA (TBE) buffer [40 mM Tris-HCl, 20 mM Boric acid (Sigma-Aldrich, USA) and 1 mM EDTA (Sigma-Aldrich, USA), pH 8.0]. The bands were visualised and captured using a UVP Doc It transilluminator (Ultra-violet Products Incorporated, USA).

### Determination of the genetic relationship of clinical *A. baumannii* isolates using the PFGE assay

The Pulsenet PFGE protocol [30] was followed with some modifications. The optical density (OD) was measured at 630 nm. The absorbance was adjusted to an OD range of 1.4 to 1.6 by using the ELx800 absorbance microplate reader (Bio-TEK, USA). The plugs were incubated overnight at 51°C and after incubation the plug was transferred to a new BD falcon tube (BD Biosciences, USA). The *Apa*I enzyme (New England Biolabs, USA) was used and the *Salmonella choleraesuis* serovar Braenderup (ATCC BAA-664) was used as a marker. Gel electrophoresis was performed using the following set conditions: interval 25 linear 5; angle 120° constant; voltage 220 V linear to 200 V and ran for 24 h. After the PFGE run was completed, the 1.2 % (m/v) SeaKem® LE agarose (Lonza, USA) gel was stained in 1 L distilled water, containing 250 μl EtBr (10 μg.ml-1) for 15 min and was destained in distilled water for 30 min. The gel was viewed and documented with a UV Doc It transilluminator (Ultra-violet Products Incorporated, USA). The banding patterns were analysed using the GelCompar II software programme (Applied Maths, Belgium). A distance matrix was constructed using the Dice coefficient and a dendogramme was constructed using the unweighted pair group method with arithmetic mean (UPGMA) [31]. Pulsotype designation was based on isolates showing ≥80 % relatedness, which corresponds to the Tenover criteria of possibly related 4 to 6 band differences between isolates [32, 33]. Representatives from each major PFGE pulsotype (≥5 isolates) and selective minor pulsotypes (< 5 isolates) with ≥80 % similarity were chosen for MLST analyses.

### Molecular epidemiology

Seven housekeeping genes were used for MLST which included the following genes: (i) citrate synthase (*glt*A); (ii) DNA gyrase subunit B (*gyr*B); (iii) glucose dehydrogenase B (*gdh*B); (iv) homologous recombination factor (*rec*A); (v) 60-kDa chaperonin (*cpn*60); (vi) glucose-6-phosphate isomerase (*gpi*) and (vii) RNA polymerase sigma factor (*rpo*D) (http://pubmlst.org/abaumannii/info/primers_Oxford.shtml). All the primers were synthesised by Inqaba Biotechnical Industries, South Africa.

All seven housekeeping genes were individually amplified in a 50 μl reaction as described in the Bioline kit’s guideline (Bioline, UK). The following programme was used on the thermal cycler (G-storm Model GS4822, Labtech, UK): an initial denaturation step of 94 °C for 2 min, followed by 35 cycles of denaturation at 94 °C for 30 s, an annealing temperature at 50 °C for 30 s, extension at 72 °C for 30 s, followed by the final extension step at 72 °C for 5 min [34]. The amplicons were visualised on a 1.8 % (m/v) MetaPhor agarose gel (Lonza, USA) as described previously. All the amplicons were sequenced in both forward and reverse directions by Inqaba Biotechnical Industries, South Africa. An ABI file obtained for each sequence were analysed with the CLC Main Workbench Version 6.0 (CLCbio, USA). The sequences were assigned to corresponding allelic profiles and sequence types by using the MLST Oxford database (http://pubmlst.org/abaumannii/).

### Data analysis

One hundred consecutive *A. baumannii* isolates were collected, which were identified by the automated VITEK® 2 system (BioMérieux, France). Six isolates were excluded from this study because the isolates were incorrectly identified as *A. baumannii* by the VITEK® 2 system. These isolates were identified by MALDI-TOF analysis as: *A. pittii*; *K. pneumoniae*; *Pseudomonas putida* (2x); *Staphylococcus aureus* and *S. epidermidis*.

The results are reported on 94 clinical *A. baumannii* isolates. The proportions of *A. baumannii* isolates with Ambler classes A, B and D resistance genes were determined for the 94 isolates. The results were reported as proportions or percentages along with 95 % confidence intervals for the given sample size with an accuracy of within 10 %. The antimicrobial susceptibility and genotypic results obtained in this study were compared to a study done in 2008 in the same clinical setting [5]. The antimicrobial susceptibility profiles were also compared to the surveillance data from 12 sentinel public hospitals in South Africa (SA) [35].

## Results

### Collection of *A. baumannii* clinical isolates

Fifty-eight of the isolates were collected from male patients and 36 isolates were collected from female patients. Forty-three percent of the isolates were collected from diverse sites, 4 % from urine, 41 % from sputum and 12 % from blood cultures. The mean age of the patients was 39 years (ranging from 4 days to 77 years). *A. baumannii* was isolated from various wards but was the most prevalent in the medical and pulmonology ICU (12 %). Age and sex is not a cofounder and hence the odds ratio for area need not be adjusted. The crude odds ratio is 1.61 with a 95 % confidence interval (0.60; 4.31) and is not significant (*p* = 0.345).

### Susceptibility profiles of *A. baumannii* isolates using the VITEK 2® system

All 94 of the *A. baumannii* isolates were resistant to ampicillin, amoxicillin, cefuroxime, cefuroximine axetil, cefoxitin, cefotaxime and nitrofurantoin. The *A. baumannii* isolates showed high level carbapenem and cephalosporin resistance (imipenem 86 %; meropenem 86 %; cefepime 90 % and ceftazidime 89 %). Table 2 shows the antimicrobial susceptibility profiles of the collected 94 *A. baumannii* isolates.

**Table 2:** Antimicrobial susceptibility profiles of the 94 collected *A. baumannii* isolates

Number of isolates (*n*=94)	Antimicrobial agent																
	1	2	3	4	5	6	7	8	9	10	11	12	13	14	15	16	17
39	R	R	R	R	R	R	R	R	R	R	S	R	R	S	R	S	R
15	R	R	R	R	R	R	R	R	R	R	S	S	R	S	R	S	R
14	R	R	R	R	R	R	R	R	R	R	S	R	S	S	R	S	R
7	R	R	R	R	R	R	S	S	S	S	S	S	S	S	R	S	S
5	R	R	R	R	R	R	R	R	R	R	R	R	R	S	R	S	R
2	R	R	R	R	R	R	R	R	R	R	S	I	R	S	R	S	R
2	R	R	R	R	R	R	R	R	R	R	S	S	R	I	R	S	R
2	R	R	R	R	R	R	R	R	R	R	I	R	R	S	R	S	R
1	R	R	R	R	R	R	S	S	S	S	S	S	S	S	R	S	R
1	R	R	R	R	R	R	S	S	S	S	S	S	I	S	R	S	S
1	R	R	R	R	R	R	R	R	S	S	S	R	R	S	R	S	R
1	R	R	R	R	R	R	R	R	R	R	R	S	I	S	R	S	R
1	R	R	R	R	R	R	R	R	I	I	R	R	R	S	R	S	R
1	R	R	R	R	R	R	R	R	R	R	S	R	I	S	R	S	R
1	R	R	R	R	R	R	R	R	S	S	S	S	R	S	R	S	R
1	R	R	R	R	R	R	I	R	S	S	S	S	I	S	R	S	S

R = resistant; S = susceptible; I = intermediate resistant

Antimicrobial agents: 1 = Ampicillin; 2 = Amoxicillin/Clavulanic acid; 3 = Cefuroxime; 4 = Cefuroximine Axetil; 5 = Cefoxitin; 6 = Cefotaxime; 7 = Ceftazidime; 8 = Cefepime; 9 = Imipenem; 10 = Meropenem; 11 = Amikacin; 12 = Gentamicin; 13 = Ciprofloxacin; 14 = Tigecycline; 15 = Nitrofurantoin; 16 = Colistin; 17 = Trimethoprim/Sulfamethoxazole

**Fig. 1:**
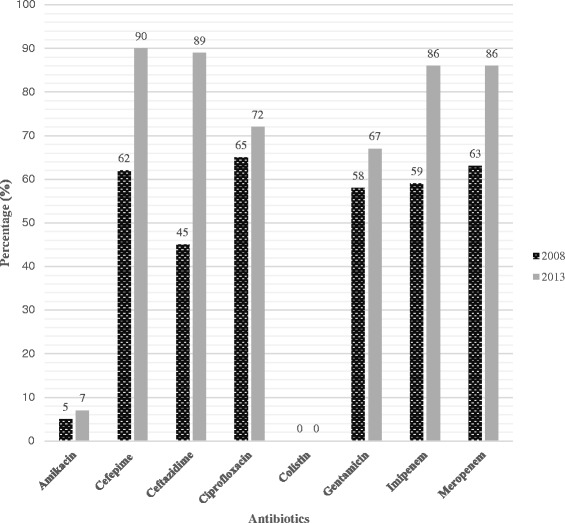
The percentage of antibiotic resistance in clinical *A. baumannii* isolates in 2008 and 2013 in the Pretoria region

The 94 *A. baumannii* isolates showed an increase in antimicrobial resistance profiles compared to the study done by Kock et al. [5] in 2008: imipenem were 59 % in 2008 and 86 % in 2013 and meropenem were 63 % in 2008 and 86 % in 2013. The cephalosporin resistance patterns changed as follows: cefepime were 62 % in 2008 and were 90 % in 2013; ceftazidime were 45 % in 2008 and 89 % in 2013. Figure 1 shows the overall percentage of resistance in 2008 and 2013 in the Pretoria region. Data from the communicable diseases surveillance bulletin [35] showed that the *A. baumannii* isolates collected in 2013 in public hospitals in SA have resistance towards carbapenems and cephalosporins (imipenem 77 %; cefepime 80 % and ceftazidime 73 %).

### Molecular identification of resistance genes found in *A. baumannii* isolates using M-PCR assays

**Fig. 2:**
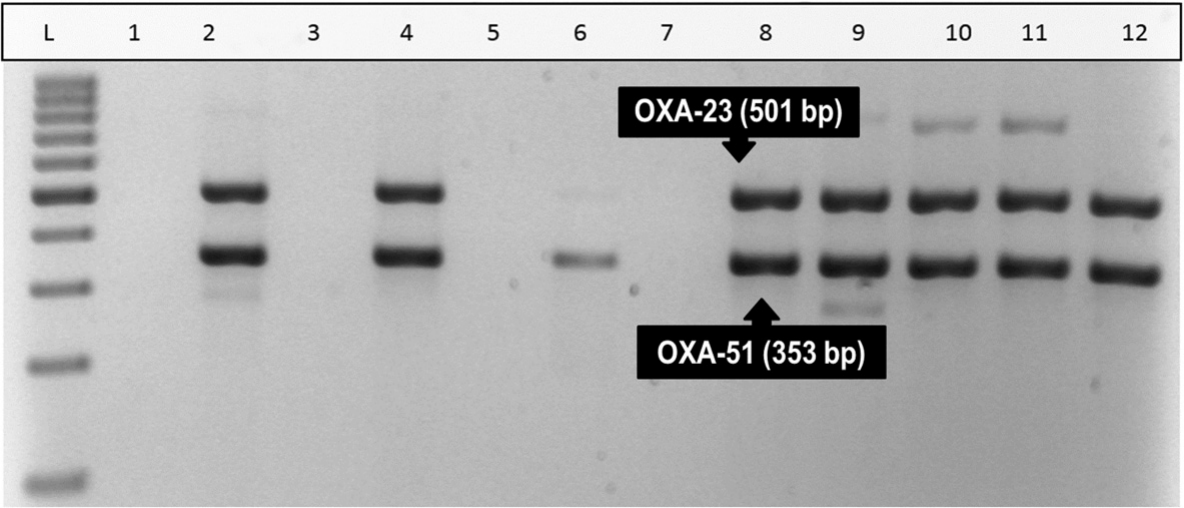
Results obtained after the M-PCR I assay, using a 1.8% (m/v) Metaphor agarose gel electrophoresis for the detection of oxacillinase (OXA) genes in *A. baumannii* isolates. The M-PCR assay amplified the OXA-23 (501 bp) and OXA-51 (353 bp) genes. Lanes 3, 5 and 7 were negative for all of the OXA genes. Lanes 2, 4, 8, 9, 10, 11 and 12 were positive for OXA-23 and OXA-51. Lane 6 was positive for OXA-51 only. Lane 1 was the negative control. Lane “L” represented the O’Range Ruler™ 100 bp DNA ladder

The M-PCR I assay showed that 99 % of the isolates contained the OXA-51 gene and 77 % contained the OXA-23 gene. None of the isolates were positive for OXA-24/40 and OXA-58. Figure 2 illustrates the M-PCR I assay, which targeted the OXA-23 (501 bp), OXA-24/40 (246 bp), OXA-51 (353 bp) and OXA-58 (599 bp) genes.

The results of the M-PCR assay I revealed the presence of two resistant profile types of *A. baumannii* that were circulating in the studied clinical setting. Type 1 was positive for both OXA-51 and OXA-23 (77 %) and type 2 was only positive for OXA-51 (22 %).

The M-PCR assay II [Imipenem metallo-β-lactamase (IMP), KPC and Verona integrin-encoded metallo-β-lactamase (VIM) genes]; III [Guyana extended-spectrum β-lactamase (GES), *Pseudomonas* extended resistance (PER) and Vietnam extended-spectrum β-lactamase (VEB) genes] and IV [German imipenemase (GIM), NDM-1, Seoul imipenem metallo-β-lactamase (SIM-1) and Sao Paulo metallo-β-lactamase (SPM) genes] were negative for all the tested genes.

### Determination of the genetic relationship and global epidemiology of clinical *A. baumannii* isolates

**Fig. 3:**
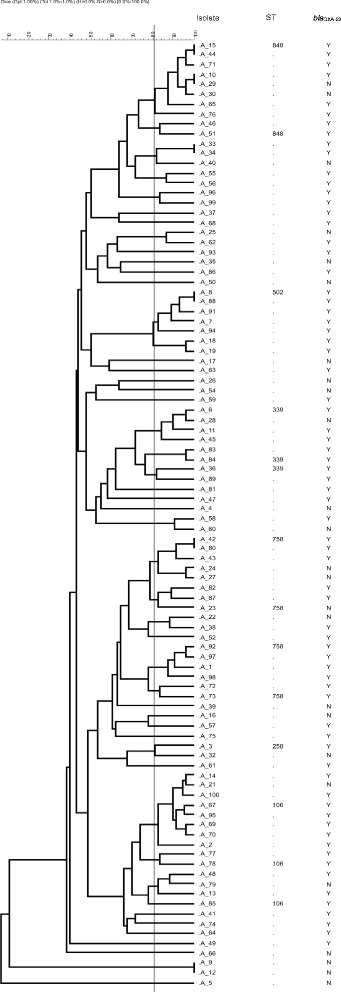
PFGE patterns of *A. baumannii* isolates. The bandings patterns were analysed using the GelCompar II software programme (GelCompar II, Applied Maths, Belgium). A distance matrix was constructed using the Dice coefficient and a dendogramme was constructed from the distance matrix using UPGMA. Major pulsotype designation was based on five or more isolates showing ≥80 % relatedness. Isolate name, ST and the carriage of *bla*_OXA-23_ (Y, positive; N, negative) are indicated

The PFGE analysis showed that the 94 *A. baumannii* isolates can be grouped into four major pulsotypes (includes ≥ 5 isolates) and several minor pulsotypes (< 5 isolates) (Fig. 3). The representative *A. baumannii* isolates from each major pulsotype and selected minor pulsotypes were grouped into five existing STs (ST106, 1-1-1-1-1-98-6; ST258, 1-15-8-10-28-110-32; ST339, 44-73-4-11-44-121-4; ST502, 1-12-3-2-2-100-3; ST758, 1-17-8-10-28-106-32 and ST848, 1-15-3-2-2-142-3). Two groups (EUI: ST258, ST758; EUII: ST502, ST848) and singletons (ST106, ST339) which shared five out of seven alleles were identified with BURST analysis (http://pubmlst.org/perl/bigsdb/bigsdb.pl?db=pubmlst_abaumannii_oxford_seqdef&page=listQuery&scheme_id=1&set_id=1).

## Discussion

The increase in resistance profiles of *A. baumannii* can be due to the misuse of antibiotics in clinical settings, or because of patients who do not complete the prescribed antibiotic course. The *bla*_OXA_ genes have an impact on treatment because it can already be resistant to the new inhibitors, such as β-lactamase inhibitors, or it can rapidly gain resistance [19].

The ability of the OXA β-lactamases to confer carbapenem resistance has already had a huge impact on the ability to treat Gram-negative pathogens and the current situation suggests that the problem is only set to increase [19]. The advantage of using molecular tests, such as the M-PCR assays used in this study, is that resistance genes circulating in clinical settings can be determined as this is not possible with phenotypic tests. Through molecular methods this study has indicated that 99 % of the *A. baumannii* isolates contained the OXA-51 gene, compared to Kock et al. [5] where only 81% out of the 97 isolates contained this gene. In the Kock et al. [5] study, the species identification obtained by the VITEK® 2 system was not confirmed by an alternative method, such as MALDI-TOF MS and thus possibly includes the other species of the *A. baumannii-calcoaceticus* complex. The results obtained in the current study shows the establishment of the species, *A. baumannii* in our clinical setting. The OXA-51 cluster is chromosomally encoded and is inherent to all *A. baumannii* isolates [4, 16, 19, 23, 35]*.* Seven isolates (of which six isolates were excluded) did not have the inherent OXA-51 gene. These isolates were further analysed using MALDI-TOF, which identified the seven isolates as: *A. baumannii*; *A. pittii; K. pneumoniae; Pseudomonas putida* (2x)*; Staphylococcus aureus* and *S. epidermidis*. Studies have shown that the detection of the *bla*_OXA-51-like_ gene can be used as a simple and reliable way of identifying *A. baumannii* [36, 37]. Although it is clear that *bla*_OXA-51-like_ genes are present in at least the vast majority of isolates of *A. baumannii*, there has been some debate as to whether they are present in all isolates of this species [36]. Zander et al. [38] reported that three *A. baumannii* isolates did not harbour the predicted *bla*_OXA-51-like_ gene (353 bp) and instead amplified PCR products of 1.2 kb and 1.6 kb. The authors then used external primers and the amplicons revealed that the one isolate (Ab-511) possessed the *bla*_OXA-51_ variant *bla*_OXA-66_, which was interrupted by the insertion sequence IS*Aba*15 at nucleotide number 435, while the two other isolates (Ab-508 and Ab-653) possessed the *bla*_OXA-51_ variant *bla*_OXA-78_, which was disrupted at nucleotide number 379 by a novel insertion sequence IS*Aba*19 [38]. Further investigation of the genomic environment of the isolate identified as *A. baumannii* by MALDI-TOF will reveal the changes in the OXA-51 genetic structure. Additionally the 16S-23S ribosomal spacer region can be sequenced for further species confirmation [37].

The occurrence of the OXA-23 gene has been reported since 1985 in *A. baumannii* isolates and has now disseminated worldwide [5, 19, 39, 40]. The prevalence of OXA-23 in this study was 77 %, compared to Kock et al. [5], who reported a 59 % prevalence in the same clinical setting in 2008. It is clear that the prevalence of OXA-23 in *A. baumannii* isolates in this clinical setting is increasing. This increased occurrence can be due to poor infection control practices, spread through contact with healthcare workers or from patient to patient and prolonged antibiotic use [11]. Stricter adherence to infection prevention and control policies by healthcare workers are needed to prevent further dissemination [4, 41, 42]. Correct use of disinfectants and solutions containing ethanol is particularly important because if applied at very low concentrations *A. baumannii* bacterium can become more pathogenic [41].

The high prevalence of OXA-23 can be due to the acquisition of genetic elements, such as plasmids and transposons [1, 42]. Liakopoulos et al. [43] conducted a study in 2010 to 2011 and reported a 95 % prevalence of OXA-23 in Greece. The OXA-23 cluster can be located on a plasmid or chromosome [5]. Mugnier et al. [44] reported in 2010 that eleven isolates had the *bla*_OXA-23_ gene on the chromosome, nine isolates had the *bla*_OXA-23_ gene on a plasmid and one isolate had two copies of the *bla*_OXA-23_ gene, one on the plasmid and one on the chromosome. The OXA-23 enzyme has a higher affinity to hydrolyse imipenem than meropenem, ertapenem or doripenem [19]. The MDR *A. baumannii* isolates in this study that contained the OXA-23 gene showed a 69 % resistance to imipenem (1 % had intermediate susceptibility and 6 % were susceptible to imipenem). Imipenem susceptibility may be explained by the absence of the IS*Aba*I element upstream of the *bla*_OXA-23_ gene [45]. Twenty-two percent of the isolates were positive for the OXA-51 gene only. However, 17 % of these isolates were resistant to imipenem while 5 % were susceptible.

The prevalence rates of isolates that contained both the OXA-23 and OXA-51 genes were 77 %. It is evident that the carbapenem-hydrolysing class D β-lactamases (CHDL’s), such as OXA-23 and OXA-51 were prevalent in clinical *A. baumannii* isolates in this study. The high prevalence of the OXA-51 and OXA-23 group (77 %) is due to the spread and establishment of several successful clones in our clinical setting.

None of the clinical *A. baumannii* isolates contained the genes that were screened for in M-PCR II to IV as well as the OXA-24 and OXA-58 genes that were part of M-PCR I. The GIM, SIM and SPM metallo-β-lactamase (MBL) genes are rarely found in *A. baumannii* isolates [46]. Kock et al. [5] reported one *A. baumannii* isolate that was positive for the VIM-like gene. Safari et al. [47] reported that 99 % of the isolates in ICU wards of three educational hospitals of Hamadan City in Iran produced metallo-β-lactamases. In 2013, the first outbreak of NDM-1 producing *A. baumannii* was reported in France, which occurred in a surgical ICU [48]. Fortunately NDM-1 in *A. baumannii* has not yet been detected in our clinical setting. The NDM-1 gene encodes an MBL carbapenemase that has a high hydrolytic activity for carbapenem antibiotics, and much more [49].

PFGE is suited for studying isolates from a defined temporal and spatial epidemiologic setting, whereas MLST has an advantage in defining clonal lineages of isolates from larger geographic areas over time [50]. Three clonal lineages of *A. baumannii*, commonly referred to as the pan-European clonal lineages (EUI, EUII, and EUIII), have predominated in many European countries since the 1990s [50]. Isolates belonging to the pan-European clonal lineages I and II (EUI and EUII) were identified in our clinical setting. EUI (ST258 and ST758) was predominant together with the OXA-23 gene. Other diverse clones, such as ST106 and ST339 are also circulating in our clinical setting. The origin of the *A. baumannii* isolates in our setting cannot be attributed to the dissemination of one or two successful clones but rather to the dissemination of a diverse group of successful clones.

## Conclusions

The increasing resistance to carbapenems are of great concern due to the limited number of effective drugs and the association with increased morbidity and mortality. There was a noticeable change in the resistance profiles of the *A. baumannii* isolates from 2008 to 2013 in the same setting. The high prevalence of MDR *A. baumannii* isolates has a severe impact on treatment outcomes in the studied healthcare facilities. The presence of the OXA-23 gene was not related to a specific ST and can be due to the acquisition of genetic elements, such as plasmids and transposons. OXA-23 and ST758 were dominant among the isolates. A limitation of this study is the small sample size and only tertiary academic hospitals in the Pretoria region were included and thus the findings in this study are not a true reflection of the situation in the whole of SA. Another limitation of this study is that there were not any positive controls included for the screened genes in M-PCR assay III, which could have caused that these genes were missed due to not using the optimal PCR conditions.

It is vital to identify and characterise healthcare-associated pathogens and their circulating antibiotic resistance genes as it is of great concern to public health systems. Understanding the resistance genes can lead to the understanding of their origin and can assist in better infection control measures and ultimately limit the spread of these pathogens.

## Abbreviations

AmpC: Ampicillin class C
CHDL: Carbapenem-hydrolysing class D β-lactamase
EtBr: Ethidium bromide
GES: Guyana extended-spectrum β-lactamase
GIM: German imipenemase
ICU: Intensive care unit
IMP: Imipenem metallo-β-lactamase
KPC: *Klebsiella pneumoniae* carbapenemase
MALDI-TOF: Matrix assisted laser desorption ionization-time of flight mass spectrometry
MBL: Metallo-β-lactamase
MDR: Multidrug resistant
MLST: Multilocus sequence typing
M-PCR: Multiplex polymerase chain reaction
NDM: New Delhi metallo-β-lactamase
NHLS: National health laboratory services
NRF: National Research Foundation
OD: Optical density
OXA: Oxacillinases
PBS: Phosphate buffered saline
PER: *Pseudomonas* extended resistance
PFGE: Pulsed field gel electrophoresis
SA: South Africa
SIM: Seoul imipenem metallo-β-lactamase
SPM: Sao Paulo metallo-β-lactamase
ST: Sequence type
TBE: Tris-Borate-EDTA
TSB: Tryptone soya broth
VAP: Ventilator-associated pneumonia
VEB: Vietnam extended-spectrum β-lactamase
VIM: Verona integrin-encoded metallo-β-lactamase
